# Endometrial cancer: an overview of novelties in treatment and related imaging keypoints for local staging

**DOI:** 10.1186/s40644-018-0180-6

**Published:** 2018-12-04

**Authors:** Stefania Rizzo, Marco Femia, Valentina Buscarino, Dorella Franchi, Annalisa Garbi, Vanna Zanagnolo, Maria Del Grande, Lucia Manganaro, Sarah Alessi, Caterina Giannitto, Francesca Ruju, Massimo Bellomi

**Affiliations:** 1 Department of Radiology, Istituto Europeo di Oncologia, via Ripamonti 435, 20141 Milan, Italy; 2 Università degli Studi di Milano, Postgraduation School in Radiodiagnostics, Via Festa del Perdono 7, 20122 Milan, Italy; 3 Department of Gynecologic Oncology, Istituto Europeo di Oncologia, via Ripamonti 435, 20141 Milan, Italy; 4 Oncology Institute of Southern Switzerland, San Giovanni Hospital, 6500 Bellinzona, Switzerland; 5 Dipartimento di Medicina Interna e Specialità mediche, Università degli Studi di Roma La Sapienza, Roma, Italy; 6 Department of Oncology and Hemato-Oncology, Università degli Studi di Milano, via Festa del Perdono 7, 20122 Milan, Italy

**Keywords:** Endometrial cancer, Myometrial invasion, Cervical invasion, MR, TVUS, DWI

## Abstract

Endometrial cancer is the most common gynaecologic malignancy in developed countries and its incidence is increasing. First-level treatment, if no contraindicated, is based on surgery. Pre-operative imaging is needed for evaluation of local extent and detection of distant metastases in order to guide treatment planning. Radiological evaluation, based on transvaginal ultrasound, MR and CT, can make the difference in disease management, paying special attention to assessment of entity of myometrial invasion, cervical stromal extension, and assessment of lymph nodal involvement and distant metastases.

## Background

Endometrial cancer (EC) is the most common gynaecologic malignancy in developed countries and its incidence is increasing. It usually arises in post-menopausal women although 20–25% of ECs are diagnosed before menopause [1]. Multiple risk factors are involved in the development of EC including obesity, unopposed oestrogen, early menarche and late menopause.

EC is traditionally classified into two major types (I and II). Type I tumours (G1-G2 endometrioid adenocarcinomas) account for about 80% of endometrial carcinomas, have a favourable prognosis and are oestrogen-responsive. Type II tumours account for 10–20% of EC, include high grade endometrioid tumours, and non-endometrioid tumours (serous, clear-cell, mucinous, squamous, transitional cell, mesonephric, carcinosarcoma, and undifferentiated), have a poor prognosis and are not clearly associated with oestrogen stimulation [2].

Staging of EC is based on the FIGO (International Federation of Gynecology and Obstetrics) staging system [3] and on TNM categories, which refer to surgical results. The standard of care for early stage EC patients with a good performance status and resectable tumour has been based on surgical removal of uterus and adnexa +/− pelvic and lombo-aortic lymphadenectomy. Pre-operative imaging by US, MR and CT has to specifically focus on evaluation of local extent (myometrial and cervical invasion), and detection of nodal and distant metastases, in order to assess prognosis and to help treatment planning.

## Main text

### Novelties in treatment planning

In 2009 the FIGO staging of EC has been updated, and FIGO stage Ia and Ib have been merged, because it has been demonstrated that the prognosis for tumor confined to endometrium or tumor invading the inner half of the myometrium was comparable [3], thus reducing the subgroups of stage I to Ia (EC confined to endometrium or invading the inner half of the myometrium) and Ib (EC invading the outer half of the myometrium).

Furthermore, it has become more and more evident that prognosis of EC patients depends on multiple different factors, most of which not included in the FIGO staging, such as histological type and grade, age of the patient, size of the tumour, lymphovascular spaces invasion. Therefore, on one hand many multicentric trials and society guidelines have attempted to include multiple prognostic factors in classification of EC [4]; on the other hand, recent advances in molecular classification of EC by histology has raised the evidence that different outcomes may be related to different expression of specific mutations and alterations, such as TP53, PI3K, KRAS, ERBB, FGFR and others [4].

Additionally, there has been a long debate about performance of lymphadenctomy. Indeed, lymph nodal involvement is the most common form of extrauterine disease spread, related to deep invasion (> 50%) of the myometrium, grade (G3) or non-endometrioid histologies [5], and is the strongest predictor of recurrence. After conflicting results, such as those of two randomized trials, showing no therapeutic benefit of lympadectomy [6, 7], and a retrospective study showing a better overall survival rate in intermediate and high-risk EC patients who underwent systematic lymphadenectomy [8], sentinel lymph node mapping has been proposed as surgical technique to assess lymph nodal status. This represents an innovative technique to identify lymph node metastases, while reducing the surgical morbidity (lymphoedema, lymphocysts) associated with systematic lymphadenectomy [9]. It has to be noted that some centres, especially in the US, thanking also the advent of the sentinel node mapping during surgery, do not perform routinely MR for pre-operative staging. However, MRI is specifically recommended during the initial workup of EC patients, in the US as well as in Europe, to establish the origin of a tumor (endocervical or endometrial), as well as when planning a fertility-sparing treatment, in order to confirm the initial stage by excluding myometrial invasion, as well as adnexal or pelvic nodes involvement [2, 9].

Finally, minimally invasive surgery, including robotic assisted surgery, has been introduced, as associated with significantly fewer moderate-to-severe postoperative adverse events (14% versus 21%) and a lower frequency of hospitalisations > 2 days (52% versus 94%) than laparotomy [2, 10]. For surgical planning, an important factor not significantly changed overtime, is cervical stromal invasion, that may require radical hysterectomy instead of total hysterectomy (performed in patients with tumours confined within the uterus [11]), which may represent a helpful information for planning operating time, especially with minimally invasive techniques.

### Novelties in imaging evaluation

The most common initial symptom (75%) of EC in postmenopausal women is abnormal uterine bleeding, and this allows the possibility of making an early diagnosis.

Gynaecological examination is usually completed by transvaginal ultrasound (TVUS), which usually represents the first imaging evaluation. TVUS is performed with a dedicated vaginal probe using high frequencies and a small field of view, which allows good local evaluation. Many studies indicate an endometrial thickness of 5 mm as the normal cut-off value in post-menopausal women [4], but in the presence of bleeding this cut-off value may be lowered to 3 mm [12]. Three-dimensional TVUS (3D-TVUS) is a novel technique which acquires US images that may be reconstructed in any desired plane, to better depict myometrial invasion in the uterine corners [13]. However, results are equivocal and 3D-TVUS has not yet proved superior to conventional 2D-TVUS in the assessment of myometrial involvement and identification of cervical infiltration [14]. When TVUS is performed in a referral centre, it is not always requested to perform an additional imaging examination for evaluation of local extent, such as MR, and the patient may complete the pre-operative staging with a CT scan.

MR with paramagnetic contrast agent is considered an accurate imaging technique for local staging, because the tumoral tissue, the endometrium and the myometrium show different MR signals, especially on T2w images. However, since these differences may be less evident in post-menopausal women, acquisition of dynamic images after injection of contrast medium is recommended [15]. The European Society of Uro-genital Radiology has suggested in 2009 a dedicated MR protocol for accurate local staging of endometrial carcinoma [15], based on T2-w acquisitions in three orthogonal planes oriented to the uterine cavity (sagittal, axial oblique and coronal oblique), with additional sequences oriented to the long axis of the endocervical canal. The slice thickness recommended is 3–4 mm with a small FOV of 20–25 cm. In order to detect nodal involvement, coronal T1-w with extended FOV is also suggested. Finally, para-axial dynamic T1-w after intravenous bolus injection of a paramagnetic contrast agent is advocated for optimal evaluation of myometrial invasion.

After publication of the abovementioned guidelines, many advances in knowledge of EC behaviour and in imaging technology have been achieved, with the consequence of some novelties in patient management.

In literature, several articles have been published about the role of 3 T MR in the preoperative evaluation of depth of myometrial infiltration and accuracy has been reported equivalent to that of 1.5 T MR [16]. MR imaging using 3 T equipment, with a higher Signal-to-Noise Ratio (SNR) than 1.5 T examinations, may lead to a more advanced temporal and spatial resolution, although it is also known that at 3 T the quality of abdominal imaging may be influenced by some artefacts. In fact, there are some known restrictions to the use of a higher magnetic field, such as an increased T1 relaxation time, a reduced T2 relaxation time and a higher magnetic susceptibility and chemical shift artifacts [17].

At the time of publication of these guidelines, Diffusion-Weighted Imaging (DWI) was indicated as a promising tool in assessing gynecologic cancers, providing information on tumour cellularity and microenvironment. After some years of experience, DWI is currently widely used as an adjunct to T2w and dynamic imaging in routine clinical practice [18]. Indeed, it is now well known that DWI is a helpful tool in detecting EC when endometrial biopsy is technically impossible, due to cervical stenosis, as well as when histopathologic results are inconclusive, because ADC values are significantly lower in cancer than normal endometrium or benign polyps [19, 20]. A recent systematic review and meta-analysis evaluating pre-operative staging of myometrial invasion in EC patients has shown that in different studies published between 2008 and 2013, DWI sequences were performed with different high b-values ranging between 500 and 1000, where the highest b-value (b = 1000) was mainly on 3 Tesla machines [21–23], whereas on 1.5 Tesla machines the highest b-value used was more frequently b = 800 [24, 25], with few exceptions [26].

It is also under consideration if the combination of T2w images and DWI may be superior to dynamic contrast-enhanced MR. Indeed, a meta-analysis including 9 studies (442 patients) comparing the diagnostic accuracy of dynamic contrast-enchanced (DCE) MR imaging and DWI to predict deep myometrial invasion, showed no differencies in pooled sensitivities and specificities of the two sequences [27]. However, in a more recent meta-analysis including 15 studies and 849 patients, the specificity of T2w images plus DWI was superior to that of DCE MR imaging [28].

Evidences suggest that fusion T2-DWI imaging at 3-T has an 88% accuracy in the assessment of the depth of myometrial invasion, with a similar diagnostic performance as DCE MRI [29]. In addition, the use of a reduced FOV for DWI imaging may improve the local staging accuracy of MRI in the assessment of the depth of myometrial invasion; in particular for patients with coexisting adenomyosis, it can be a helpful alternative diagnostic tool to 3D DCE MRI [30]. Field-of-view (FOV) optimized and constrained undistorted single shot (FOCUS) is a new sequence clinically applied by using a two dimensional spectral spatial excitation radiofrequency pulse, which limits the signal to a small FOV, thus excluding possible sources of artifacts that are outside the region of interest [30]. This may avoid local field homogeneity conditions, and minimize off-resonance artifacts due to B0 inhomogeneity, susceptibility gradients, eddy currents, and chemical shift [31, 32]. Reduced FOV DW imaging with high spatial resolution may enhance the lesion detection, and the evaluation of tumor extent in endometrial cancer. Indeed, severe geometric distortion at air-tissue interfaces around the uterus, ovaries and rectum, can be minimized on reduced FOV DW imaging. However, since the small FOV may be a limit for the detection of lymph node metastases, peritoneal dissemination and/or bone metastases, reduced FOV DW imaging is considered as an additional sequence to the traditional large FOV DW sequence, for the evaluation of local tumor extent [30].

Bhosale et al. reported the feasibility of reduced FOV DW imaging in the assessment of myometrial invasion in patients with clinical FIGO stage I endometrial cancer. In this study the authors demonstrated the feasibility of IVIM parameters as imaging markers of MSI status (Micro-Satellite Instability), defined as a germline mutation of mismatch repair system (DNA polymerase) that leads to a higher risk to develop EC. Furthermore, compared with T2-weighted imaging plus DCE MR, reduced FOV DW imaging yielded greater specificity and accuracy for deep myometrial invasion determined by one reader, and greater the sensitivity for deep myometrial invasion determined by another reader [31].

Recently there has been a growing need for a non-invasive imaging method for the accurate diagnosis and differentiation of endometrial malignancy. With this regard, it has been demonstrated that DW signal and ADC values can be influenced not only by molecular proton diffusion, but also by microcirculation or blood perfusion. Some Authors have reported the feasibility to study the tissue characteristics of endometrial cancer using IVIM model at 3 T and to assess their diagnostic potential [32]. In this study, ADC values derived from a mono-exponential model of DWI containing perfusion effect were demonstrated to limit the reliability of ADC in characterizing lesions [33]. The IntraVoxel Incoherent Motion (IVIM) has been used to estimate perfusion in tissues, as blood flow in randomly oriented capillaries mimics a pseudo-diffusion process. By using IVIM-based perfusion MRI, microcirculation or perfusion effects can be distinguished from true tissue diffusion, when using a sufficient b value sampling and bi-exponential curve fit analysis with the intravoxel incoherent motion (IVIM) model [34]. IVIM MRI can be considered as an extension of DWI that enables the simultaneous acquisition of both microcirculatory and diffusivity information. The capillary network lacks the spatial orientation and the molecular motion within the microcirculation; this can be viewed as “pseudo-diffusion”. This pseudo-diffusion is significant at the lower b-value range (< 200 s/mm2) and the following two quantitative perfusion parameters can be derived from analysing the steeper part of the diffusional signal decay curve: the fractional perfusion related to the microcirculation, and the pseudo-diffusion coefficient that measures the incoherent perfusion-related diffusion within the microcirculation. The primary advantage of IVIM is that it enables the simultaneous acquisition of diffusion and perfusion parameters and therefore can provide both measurements without the requirement of a further co-registration processing step [18, 35]. The perfusion characteristic differs between tissue types in the female pelvis: tumoral perfusion affects the sensitivity to chemo-radiotherapy, whereas altered vasculatures and variable levels of aniogenic factors within gynecological disease are thought to determine therapeutic success following interventional therapy. Endometrial cancer has low perfusion and high diffusion IVIM characteristics with promising potential for early non-invasive diagnosis, hence proving potentially useful for tissue differentiation.

CT has a low sensitivity (83%) and specificity (42%) in detecting and evaluating myometrial involvement, as well as in assessing cervical stromal invasion [36]. However, dual-energy CT evaluation of deep myometrial invasion has been suggested [37], and CT is widely recognized as helpful in assessing distant metastases.

PET/CT is more sensitive than CT or MR imaging for detection of nodal metastases [38, 39]. Furthermore, in patients for whom biopsy demonstrates high-risk histology, PET/CT may be used to identify unsuspected distant disease that would obviate the morbidity of a staging operation. Two meta-analyses have indeed shown that PET/CT is highly sensitive and specific for distant nodal metastasis [40–42], although a significant percentage of lymph nodes still go undetected by PET/CT. In a direct comparison of MR imaging and PET/CT for initial staging, PET/CT was found superior for detection of metastatic lymph nodes (sensitivity 70% for PET/CT vs 34% for MR imaging) [38].

Recently introduced PET/MR scanners acquire MR and PET data either simultaneously or sequentially. In gynaecologic cancer patients, 18F-FDG PET/MR protocols are intended to provide a sole examination to solve the problems of treatment planning, as well as for evaluation of prognosis and surveillance. Indeed, in simultaneous PET/MR scanners, during the PET acquisition, whole-body Dixon images, the anatomically descriptive half-Fourier acquisition single-shot turbo spin-echo images, and fluid-sensitive inversion recovery images and DWI are co-acquired; these acquisitions are then completed for local staging by a dedicated pelvic MR including dynamic intravenous gadolinium administration. MR imaging and PET are complementary in the initial staging of endometrial cancer because MR is more accurate for evaluation of local extent of the tumour, whereas PET is more accurate for distant metastases. Lesions detected on PET or DWI can be precisely localized and characterized on the conventional MR sequences, thereby allowing for improved sensitivity without a loss of specificity. In a direct comparison of fused PET/MR imaging, MR imaging, and PET/CT data, PET/MR imaging and MR imaging alone were both more accurate for local staging of endometrial cancer (T-stage accuracy 80% for PET/MR imaging or MR imaging alone vs 60% for PET/CT) [39].

For association with prognosis after therapy, both PET and MR imaging features of endometrial cancer have been evaluated [40]. The preoperative maximum SUV has been correlated with the presence of high-risk or low-risk disease after surgical staging [43]. Accordingly, tumors with high-risk features, such as deep myometrial invasion, cervical invasion, lymphovascular space involvement, and lymph node metastases, showed high maximum SUV and low minimum ADC [44].

### Imaging keypoints according to stage

#### Stage I

FIGO stage IA include tumours involving only the endometrium or < 50% of the myometrium. Stage IB represents > 50% invasion of the myometrium, which is a marker of potential lymphovascular space invasion and, therefore, nodal metastases.

**Fig. 1:**
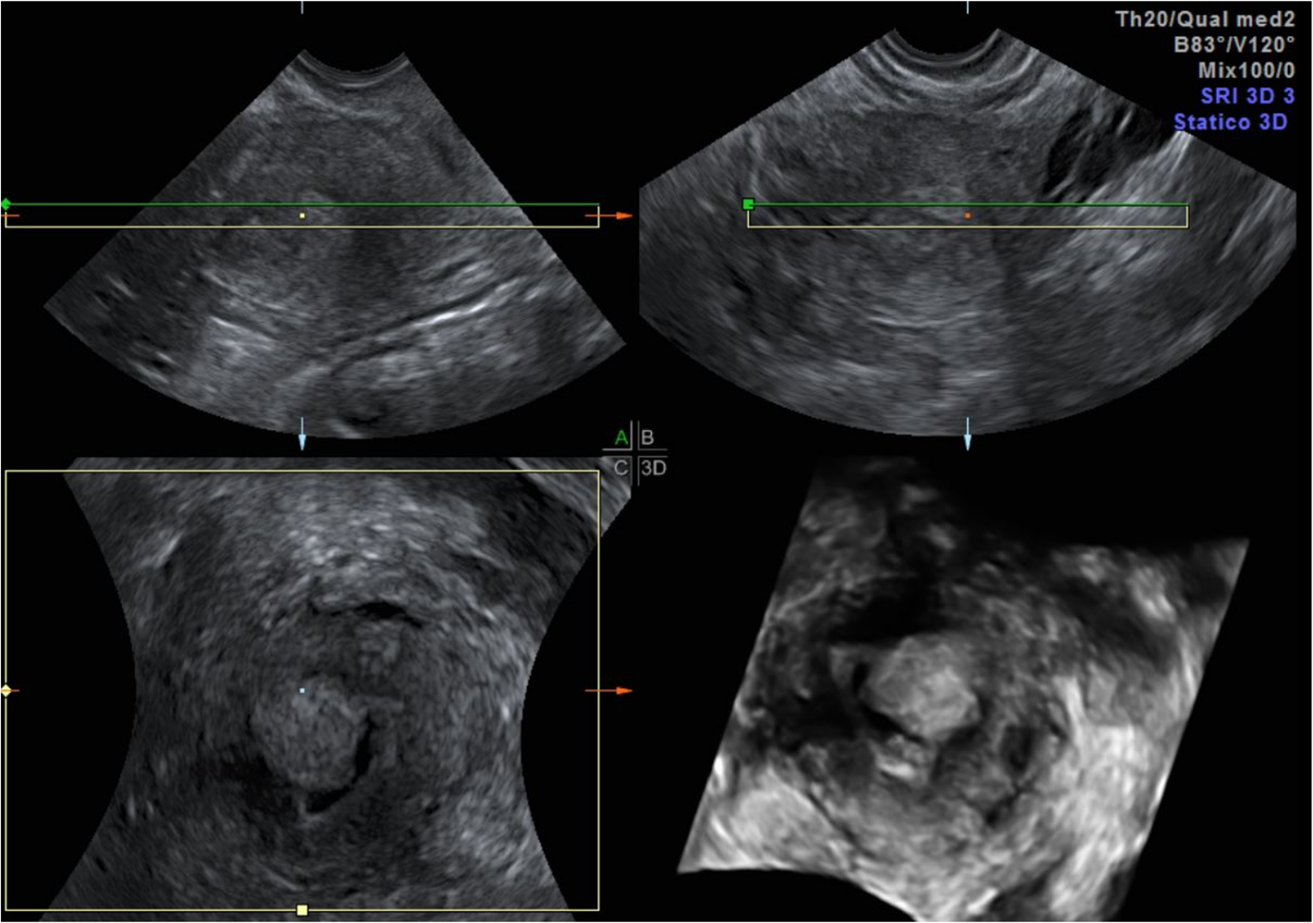
A 52-year-old woman with endometrioid adenocarcinoma (stage IA). Transvaginal ultrasound shows the endometrial cancer invading < 50% of the myometrium

At TVUS, myometrial invasion appears as an iso-hyperechoic tissue compared to the surrounding myometrium. Although myometrial invasion can often be well-appreciated, sometimes it can only be presumed according to an irregular aspect of the endomyometrial junction (Fig. 1) [45]. Both objective and subjective methods have been used to assess the depth of myometrial invasion. Objective assessment is performed by means of Gordon’s and Karlsson’s approaches. According to Gordon, deep myometrial invasion is defined as the ratio between maximum tumour depth and total myometrial thickness, whereas for Karlsson a > =50% ratio between the anteroposterior diameter of the lesion and the anteroposterior diameter of the uterus defines deep myometrial invasion [46, 47].

**Fig. 2:**
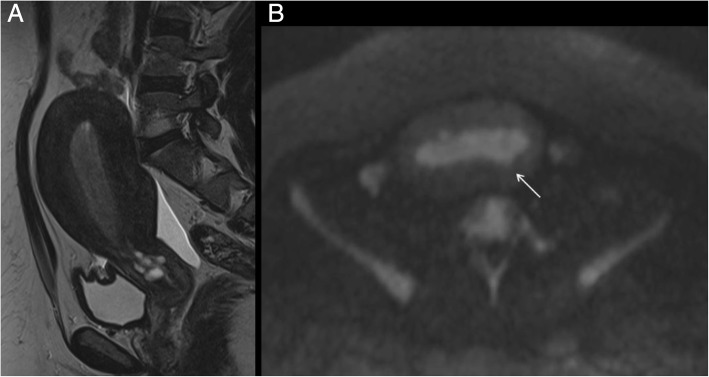
A 51-year-old woman with endometrioid adenocarcinoma invading < 50% of the myometrium. Sagittal T2-w MRI (**a**) shows an intermediate-signal intensity tumour with disruption of the low signal junctional zone but the extent of myometrial invasion is unclear. Corresponding axial DWI image (**b**) shows an area of high signal intensity within the endometrial tumour invading only the inner myometrium (arrow)

Subjective assessment of myometrial invasion (sensitivity 77%, specificity 81%) may be as good as or better than any objective measurement technique. Indeed, fixing the sensitivity at the same level as that of subjective evaluation (i.e. 77%), all objective measurement techniques (except minimal tumour-free margin/uterine AP diameter ratio) showed a significantly lower specificity [48]. MRI discriminates the degree of myometrial infiltration with a sensitivity of 87% [49]. In order to measure the depth of myometrial infiltration on MR images, a line must be drawn along the expected inner edge of the myometrium (corresponding to the endometrium–myometrium junction) on axial oblique images acquired perpendicular to the endometrium; then, 2 measures have to be taken: one representing the thickness of the entire myometrium; the other measuring the maximum tumor extent within the myometrium. The ratio of these 2 measures represent the percentage of myometrial invasion [50]. In stage IA, either the low T2 signal of the junctional zone, representing the limit between endometrium and myometrium, is normal and a complete sub-endometrial enhancement on T1 contrast imaging is present, or there is a disruption of the low T2-w signal junctional zone with < 50% invasion of the myometrium (Fig. 2).

**Fig. 3:**
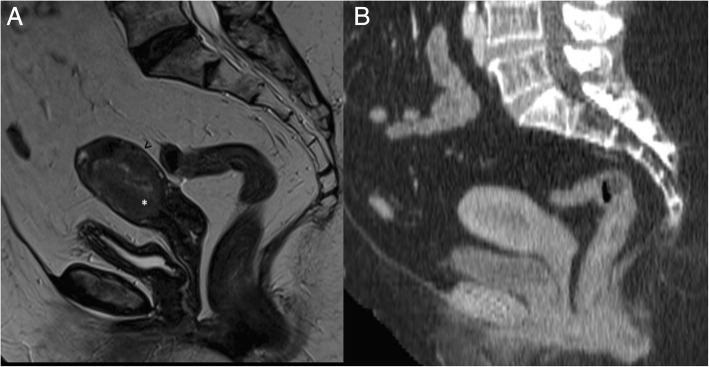
A 66-year-old woman with endometrioid adenocarcinoma, grade 3. Sagittal T2-weighted image shows an intermediate-signal intensity endometrial tumour invading > 50% of the myometrium (**a**), with disruption of the low T2 signal junctional zone (white asterisk) and preservation of the low signal band of the outer myometrium (black arrowhead). Sagittal post contrast CT (**b**) in the same patient, performed for evaluation of distant metastases, does not discriminate the extent of myometrial infiltration

**Fig. 4:**
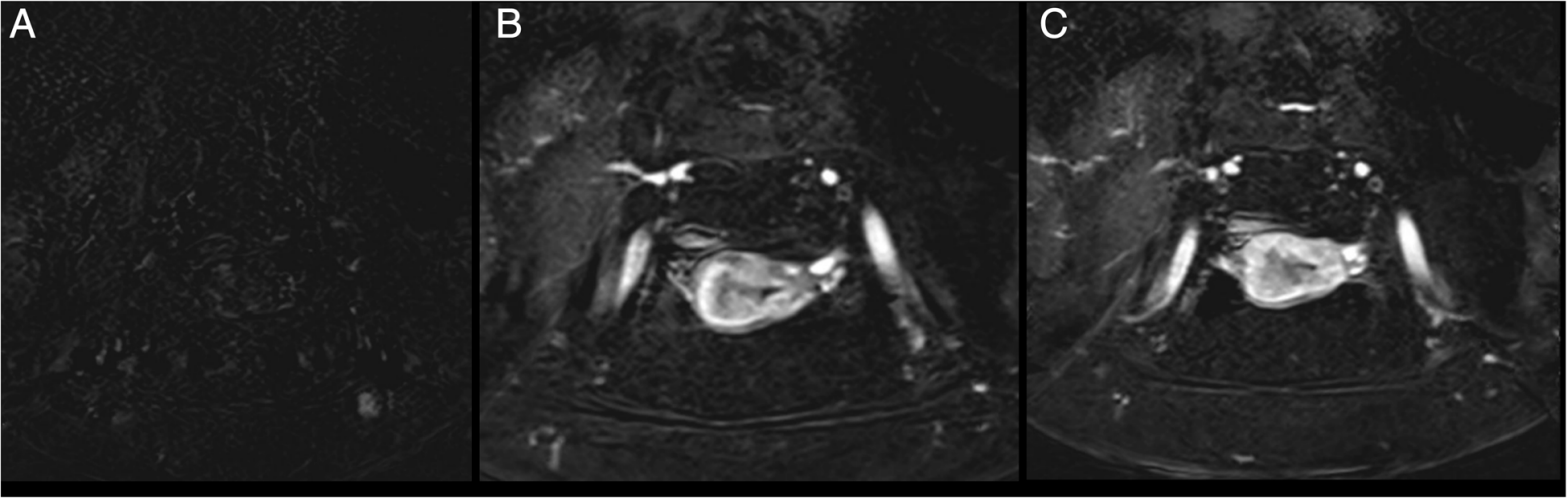
A 75-year-old woman with endometrioid carcinoma. Paracoronal post-gadoliunium T1-w subtracted dynamic images at 0 s (**a**), 60s (**b**) and 90s (**c**) help the evaluation of deep myometrial invasion in a post-menopausal patient

In stage IB tumours, disruption or irregularity of the low T2-w signal junctional zone and/or of sub-endometrial enhancement in post-contrast T1 images with myometrial invasion > 50% can be demonstrated (Fig. 3). Dynamic axial T1 images after intravenous injection of paramagnetic contrast agent with a timing between 90 and 150 s are helpful for an optimal evaluation of myometrial invasion, especially in post-menopausal women [51, 52] (Fig. 4).

**Fig. 5:**
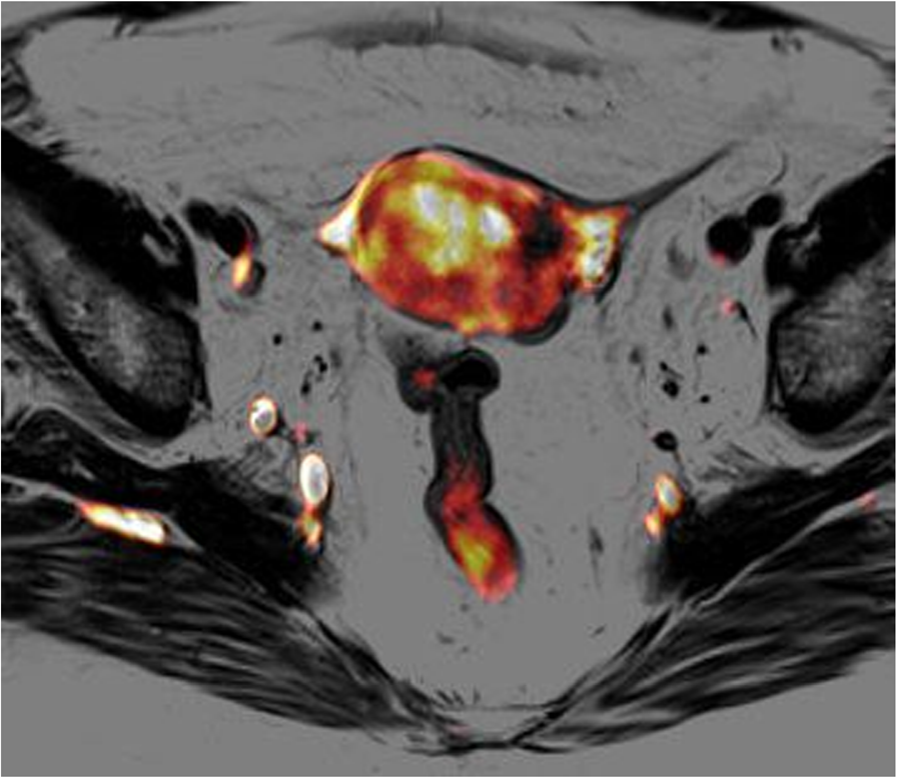
A 67-year-old patient with endometrioid carcinoma of the uterus, grade 2. Axial T2-weighted-DWI fused image shows an overlap of high DWI signal intensity and thicker uterine wall in the right side of the uterus. This makes the detection of tumour depth easier and more confident

**Fig. 6:**
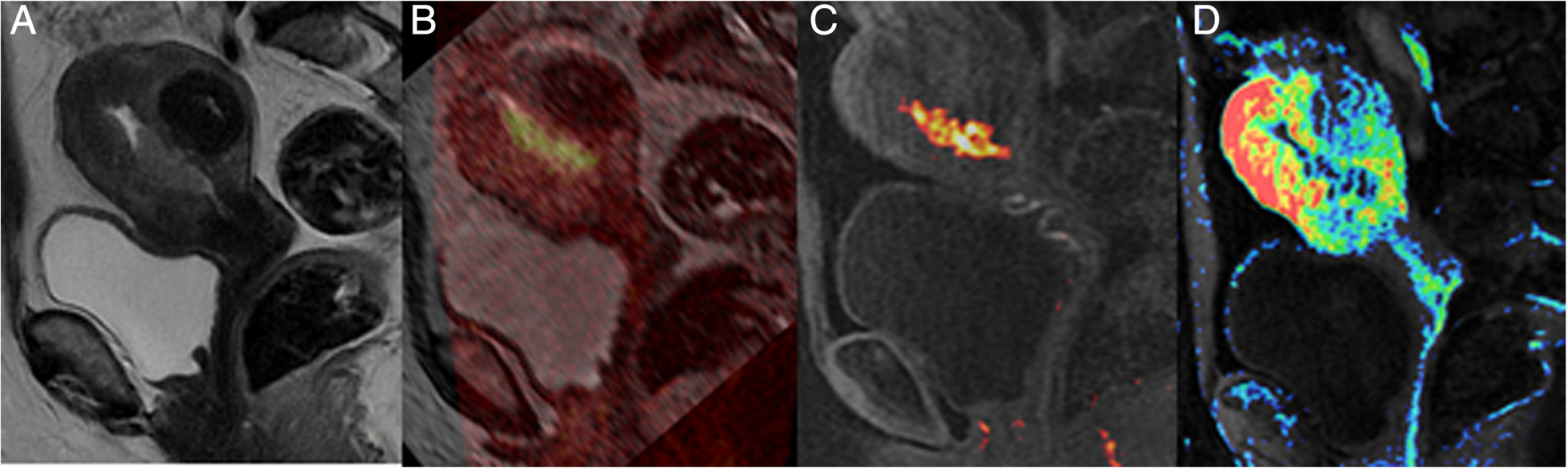
Images of a 56-year-old woman with biopsy proved prostate cancer: multiparametric study: T2WI (**a**). FOCUS DWI images of b = 1000 s/mm2 (**b** and **c**). Perfusion map T2WI on sagittal plane (**d**) shows endometrial lesion confined to the endometrial cavity. FOCUS DWI images show tumor lesion in higher spatial resolution with a definite clear border, less artifacts, and image blurring

As mentioned above, DWI sequences could help radiologists to assess myometrial invasion, alone or in combination with T2w images (Figs. 5 and 6) [29]. This might be particularly helpful in cases of tumours extending to the cornua, myometrial compression from a polypoid tumor, leiomyomas, or adenomyosis, where anatomic details on morphologic images could be confusing, or in patients with relative contraindications to gadolinium-based contrast agents.

#### Stage II

**Fig. 7:**
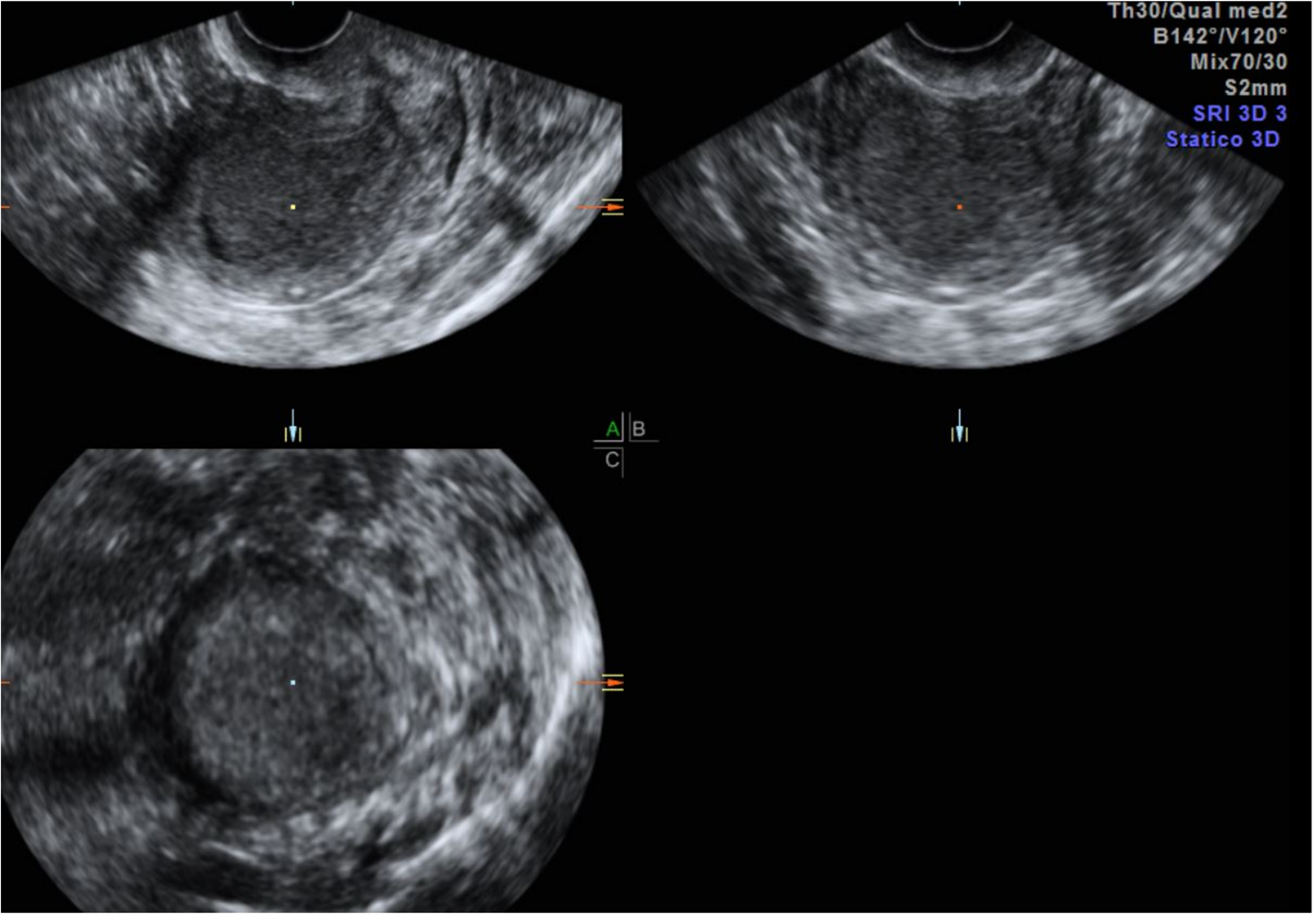
A 61-year-old women with endometrioid adenocarcinoma, grade 2. TVUS shows an exophytic isoechoic lesion extending to the cervix, as confirmed by post-surgical pathology

EC is classified as stage II in case of cervical stromal invasion, and is associated with a poorer prognosis due to the higher risk of lymphovascular space invasion. At TVUS, the assessment of cervical invasion can be difficult as prominent pathologic tissues may sometimes protrude through the internal uterine ostium into the cervical canal with no infiltration of the endocervical glandular epithelium, in which case a slight pressure with the probe may be helpful. However, iso-hyperechoic endometrial thickening of the cervical canal may suggest cervical involvement by endometrial cancer which may extend deeper into the cervical stroma (Fig. 7). The distance from the outer cervical os to the lower tumour margin is the only parameter that might have the potential to predict cervical invasion. It showed a non-significant higher sensitivity than did subjective evaluation (73% vs 54%, *P* = 0.06), but a significantly lower specificity (63% vs 93%, *P* < 0.001) [47]. Stages IIA and IIB are hardly distinguished by TVUS. Nevertheless, both groups of patients undergo radical hysterectomy, with bilateral salpingo-oophorectomy and systematic pelvic lymphadenectomy with or without para-aortic lymphadenectomy [2, 44, 53, 54].

MRI discriminates the degree of infiltration of the cervical stroma and vaginal walls with a sensitivity of 80% and, in addition, it can help in the assessment of parametrial infiltration [49].

**Fig. 8:**
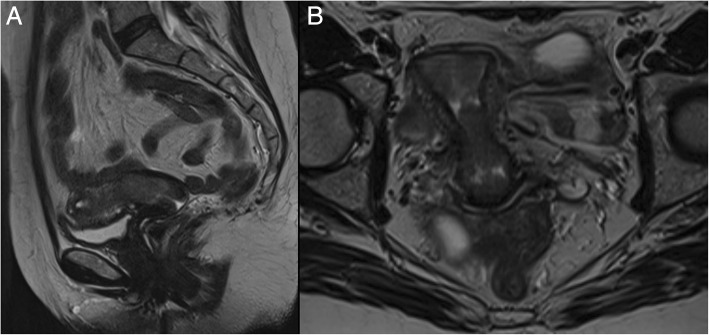
A 54-year-old women with EC. Sagittal (**a**) and axial (**b**) T2-w images show infiltration of the cervical stroma, without extension to the parametria, staging this tumour as II

In stage IIA, MR shows widening of the internal os and on T2-w images the endocervical canal is occupied by hyper/isointense tissue, with an intact low signal of the cervical stroma. If the hypointense stromal ring is disrupted, the tumour is staged as IIB (Fig. 8).

The use of dynamic contrast MRI could help distinguish between stromal invasion and polypoid tumour protruding into the endocervix [18]. If delayed phase images show tumour protrusion and normal enhancement of the cervical mucosa, stromal invasion can be excluded [55].

#### Stage III

Stage III indicates local tumour spread beyond the uterus but not outside the true pelvis. The subset IIIA shows the invasion of serosa with a disruption of the contour of the outer myometrium or adnexa involvement. The subset IIIB shows direct parametrium or vaginal infiltration. Stage IIIC indicates nodal involvement and is subdivided into pelvic (stage IIIC1) and para-aortic (stage IIIC2) nodal involvement.

TVUS can be used to diagnose metastatic disease to the ovaries with a sensitivity and specificity of subjective evaluation of grey-scale and Doppler ultrasound findings of 84–91% and 94–100%, respectively, when used by experienced ultrasound examiners with regard to making a specific diagnosis of adnexal masses [56].

**Fig. 9:**
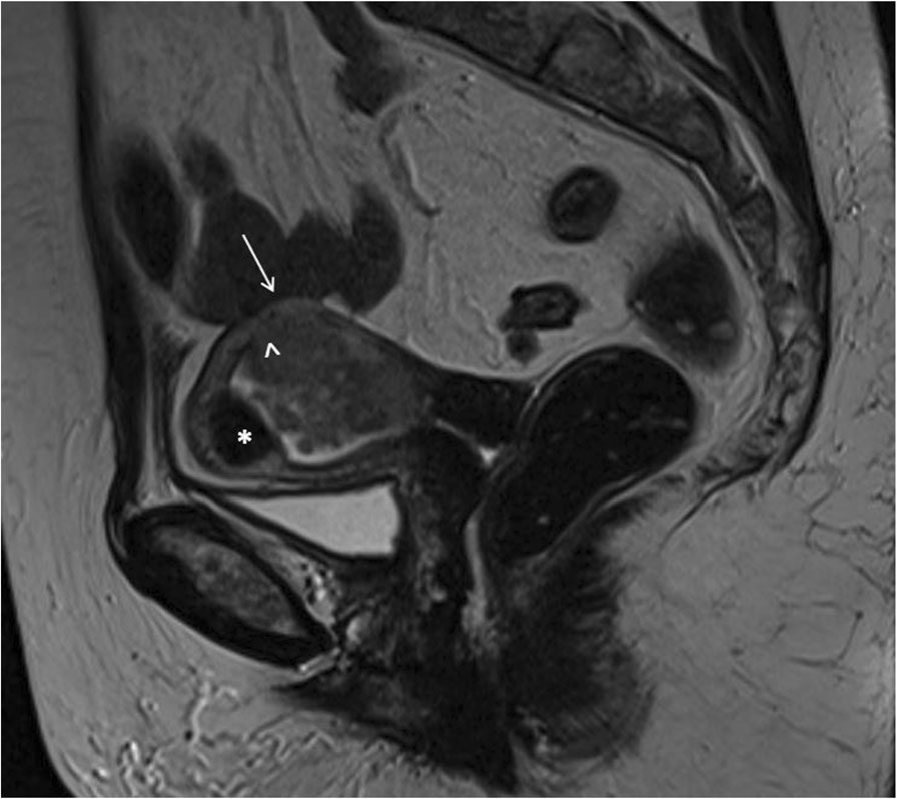
A 67-year-old woman with EC stage IIIA. MR sagittal T2-w image depicting the endometrial lesion of the posterior uterine wall extending to the internal orifice without cervical involvement. There is a disruption of the junctional zone (arrowhead) indicating myometrial invasion, and disruption of the hypointense line contouring the myometrium on the outer side of the uterus (arrow), indicating serosal invasion. Collaterally there is a submucosal leiomyoma (asterisk)

**Fig. 10:**
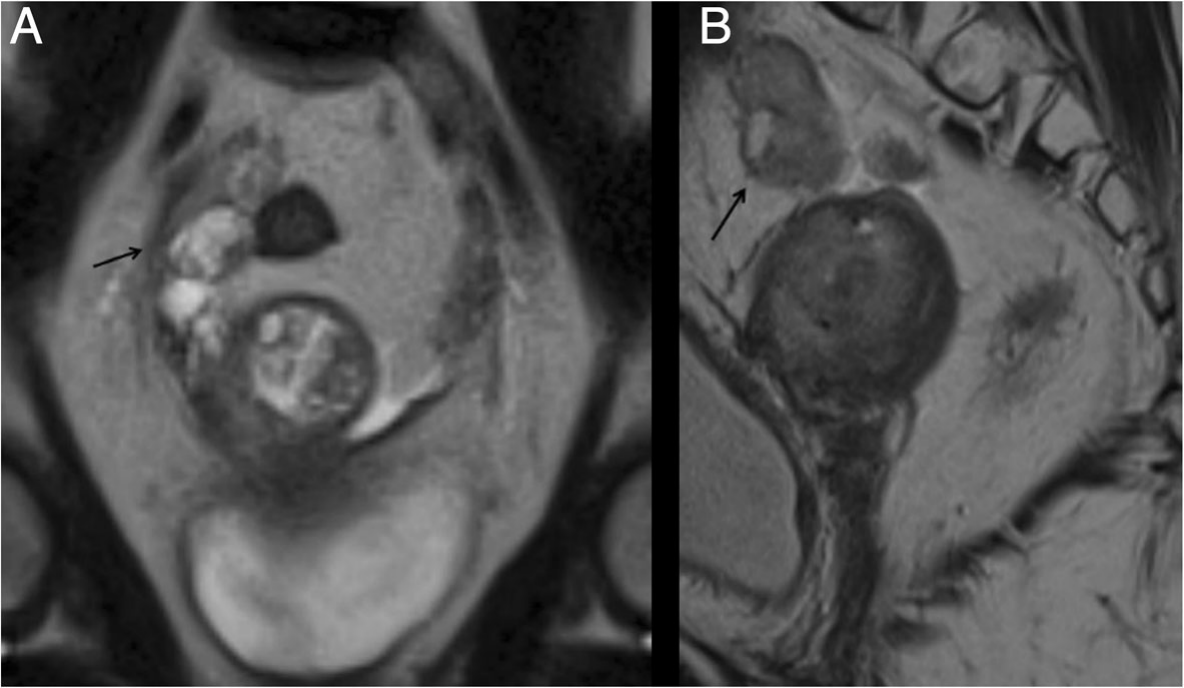
A 54-year-old EC patient with deep myometrial infiltration and serosal involvement. Coronal (**a**) and sagittal (**b**) MR images show a small mass in the right ovary (arrows) with solid components of the same signal intensity as the EC, representing ovarian involvement

**Fig. 11:**
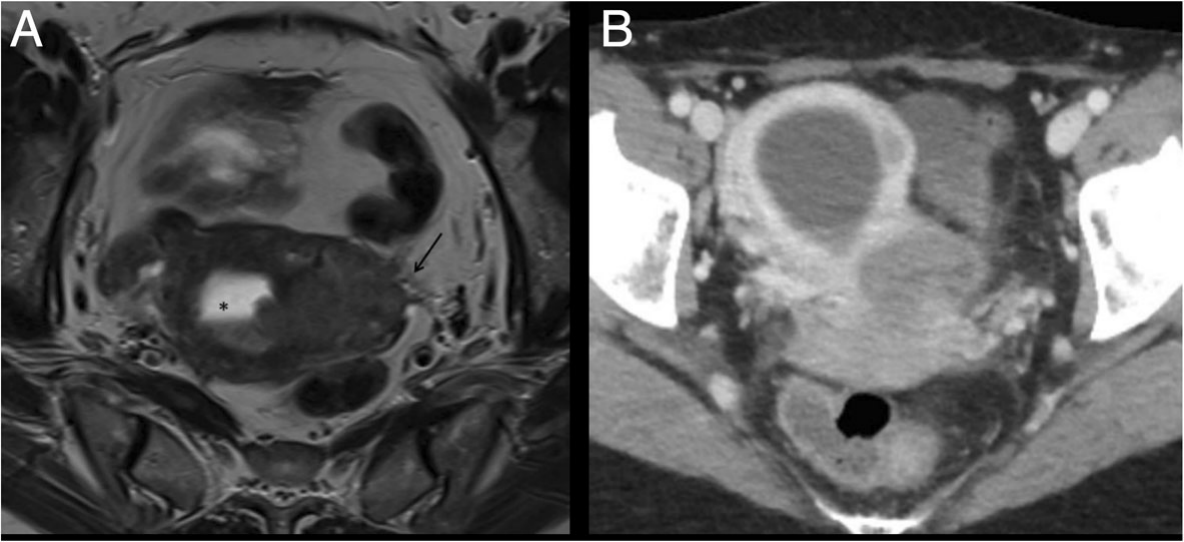
A 57-year-old woman with EC stage IIIB. Para-axial T2-w MR image (**a**) shows the endometrial carcinoma extending to the left parametrium (arrow). The tumour infiltrates the cervix through the internal uterine ostium leading to a retrodilation of the cavity which appears occupied by fluid (asterisk). In the same patient, the axial CT image shows the mass invading the left parametrium (**b**); collaterally there is a centimetric leiomyoma of the left wall of the uterus

In stage IIIA, MR shows invasion of the serosa with disruption of low T2-w signal uterine serosa (Fig. 9) and/or adnexa (Fig. 10) with irregularity to the uterine contour. In stage IIIB, MR shows thickening of the vaginal wall and a high T2-w signal tumour infiltrating the low signal vaginal wall, or irregular intensity of the parametria (Fig. 11), respectively. Also in these cases, DWI improves the depiction of extrauterine metastatic deposits into the parametrium or vagina [11].

**Fig. 12:**
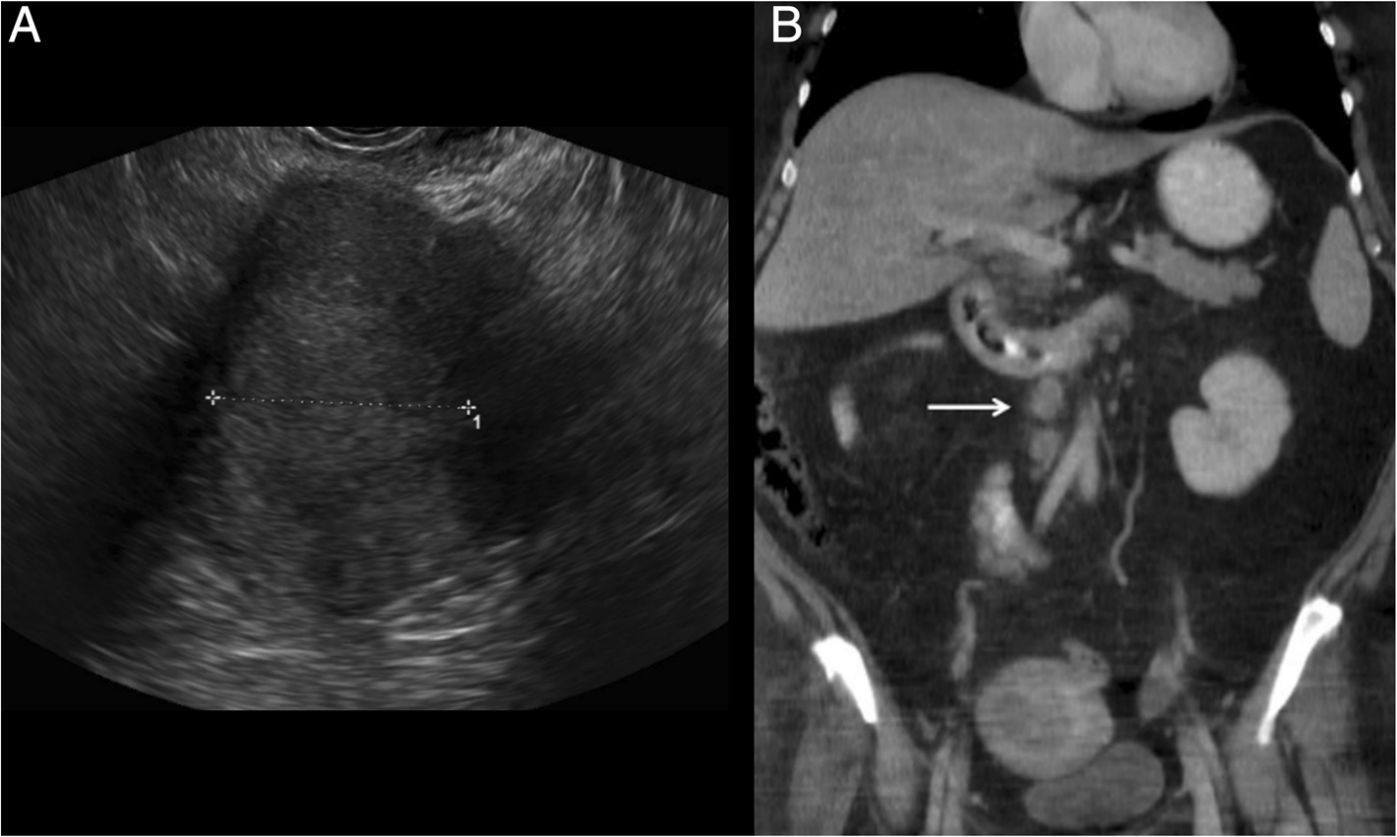
A 46-year-old woman with EC grade 3. TVUS image (**a**) shows an endometrial mass of 4.79 cm (measure in yellow) invading < 50% of the myometrium. Despite a superficial myometrial invasion, CT images at staging (**b**) showed pelvic and lombo-aortic lymphoadenopathies (arrow), that bwere confirmed at surgery

In stage IIIC, MR shows pelvic or para-aortic lymphadenopathies (Fig. 12). Further studies of 1.5-T MR images reported conflicting results [57, 58]. Lin et al. reported good results in differentiating metastatic and benign lymph nodes with 3 T-MRI, combining ADC values, relative ADC values, and size criteria [29].

#### Stage IV

**Fig. 13:**
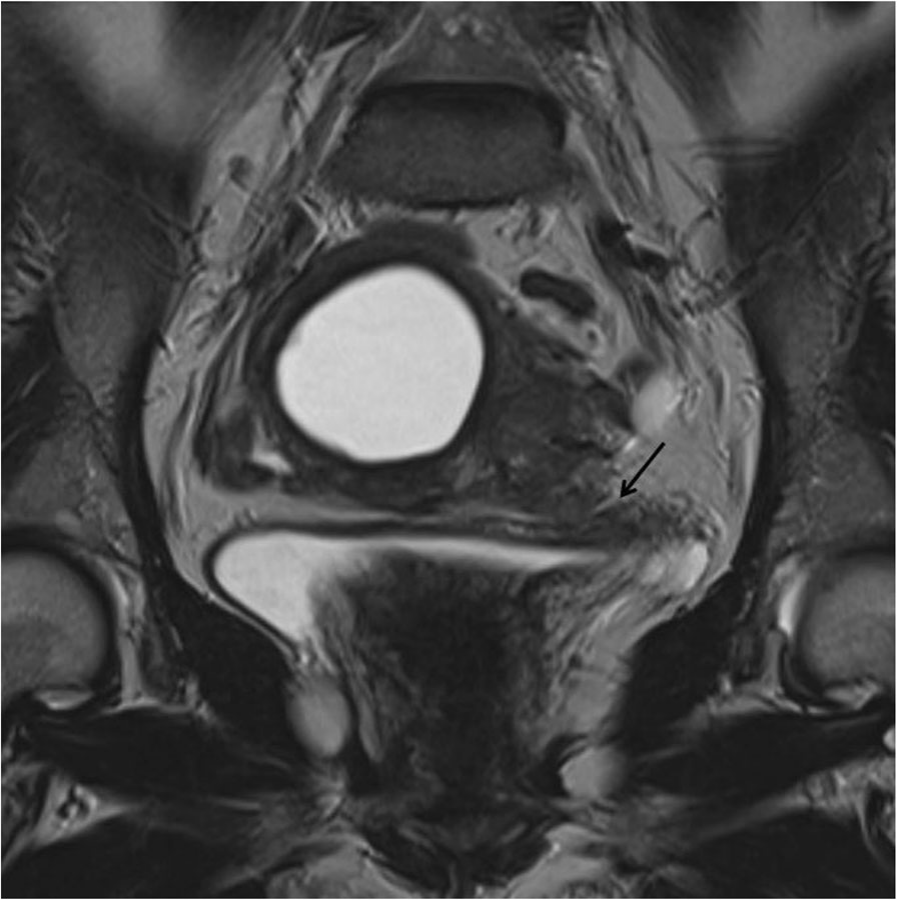
A 57-year-old woman with EC stage IVA. Coronal T2-weighted MR image showing a slightly hypointense tissue extending from the left parametrium to focally disrupt the urinary bladder wall on the left side (arrow)

Stage IV disease represents direct full thickness invasion of the bladder or rectal mucosa (stage IVA) or the presence of distant metastases (stage IVB). On T2-weighted images, extension of tumor directly into the normally hyperintense vescical or rectal mucosa is indicative of endometrial tumor invasion, however, the sole disruption of the hypointense muscularis layer does not indicate stage IV disease because it cannot be visualized at subsequent cystoscopy or sigmoidoscopy. Similarly, bullous edema, which appears as thickening of the high-signal-intensity mucosal layer, is not indicative of mucosal invasion. In stage IVA, MR may clearly depict disruption of the low T2-w signal of the bladder or rectal wall through the whole thickness of the wall, and/or intraluminal bladder mass (Fig. 13); whereas in stage IVB, malignant ascites with peritoneal deposits, para-aortic lymphadenopathy above the renal vessels or inguinal node metastases can be demonstrated.

## Conclusion

In conclusion, EC is the most common gynaecologic malignancy in developed countries. As treatment planning has been changing over time, pre-operating imaging evaluation has to follow novelties in treatment, in order to offer the appropriate information to surgeons and/or oncologists for optimal management of patients.

## Abbreviations

ADC: Apparent diffusion coefficient
DCE: Dynamic contrast enhancement
DWI: Diffusion weighted imaging
EC: Endometrial cancer
FIGO: International Federation of Gynecology and Obstetrics
FOCUS: Field-of-view Optimized and Constrained Undistorted Single shot
FOV: Field of view
IVIM: Intra Voxel Incoherent Motion
TNM: Tumour nodes and metastases staging
TVUS: Trans-vaginal ultrasound
